# Liver ERα mediates sex differences in metabolic pattern changes in response to time-restricted feeding

**DOI:** 10.1093/lifemeta/loaf011

**Published:** 2025-03-25

**Authors:** Haoqi Zhang, Tengteng Huang, Xianyang Jin, Siyuan Liu, Yi Yang, Luting Liu, Xuemei Jiang, Ruinan Zhang, Hui Ye, Xinyue Qi, Tongxing Song, Chao Jin, Bin Feng, Lianqiang Che, Shengyu Xu, Yan Lin, Zhengfeng Fang, Ting Luo, Yong Zhuo, De Wu, Lun Hua

**Affiliations:** Animal Nutrition Institute, Sichuan Agricultural University, Chengdu, Sichuan 611130, China; Key Laboratory for Animal Disease-Resistant Nutrition of the Ministry of Education of China, Sichuan Agricultural University, Chengdu, Sichuan 611130, China; Key Laboratory of Animal Disease-Resistant Nutrition of Sichuan Province, Sichuan Agricultural University, Chengdu, Sichuan 611130, China; Animal Nutrition Institute, Sichuan Agricultural University, Chengdu, Sichuan 611130, China; Key Laboratory for Animal Disease-Resistant Nutrition of the Ministry of Education of China, Sichuan Agricultural University, Chengdu, Sichuan 611130, China; Key Laboratory of Animal Disease-Resistant Nutrition of Sichuan Province, Sichuan Agricultural University, Chengdu, Sichuan 611130, China; Animal Nutrition Institute, Sichuan Agricultural University, Chengdu, Sichuan 611130, China; Key Laboratory for Animal Disease-Resistant Nutrition of the Ministry of Education of China, Sichuan Agricultural University, Chengdu, Sichuan 611130, China; Key Laboratory of Animal Disease-Resistant Nutrition of Sichuan Province, Sichuan Agricultural University, Chengdu, Sichuan 611130, China; Animal Nutrition Institute, Sichuan Agricultural University, Chengdu, Sichuan 611130, China; Key Laboratory for Animal Disease-Resistant Nutrition of the Ministry of Education of China, Sichuan Agricultural University, Chengdu, Sichuan 611130, China; Key Laboratory of Animal Disease-Resistant Nutrition of Sichuan Province, Sichuan Agricultural University, Chengdu, Sichuan 611130, China; Animal Nutrition Institute, Sichuan Agricultural University, Chengdu, Sichuan 611130, China; Key Laboratory for Animal Disease-Resistant Nutrition of the Ministry of Education of China, Sichuan Agricultural University, Chengdu, Sichuan 611130, China; Key Laboratory of Animal Disease-Resistant Nutrition of Sichuan Province, Sichuan Agricultural University, Chengdu, Sichuan 611130, China; Animal Nutrition Institute, Sichuan Agricultural University, Chengdu, Sichuan 611130, China; Key Laboratory for Animal Disease-Resistant Nutrition of the Ministry of Education of China, Sichuan Agricultural University, Chengdu, Sichuan 611130, China; Key Laboratory of Animal Disease-Resistant Nutrition of Sichuan Province, Sichuan Agricultural University, Chengdu, Sichuan 611130, China; Animal Nutrition Institute, Sichuan Agricultural University, Chengdu, Sichuan 611130, China; Key Laboratory for Animal Disease-Resistant Nutrition of the Ministry of Education of China, Sichuan Agricultural University, Chengdu, Sichuan 611130, China; Key Laboratory of Animal Disease-Resistant Nutrition of Sichuan Province, Sichuan Agricultural University, Chengdu, Sichuan 611130, China; Animal Nutrition Institute, Sichuan Agricultural University, Chengdu, Sichuan 611130, China; Key Laboratory for Animal Disease-Resistant Nutrition of the Ministry of Education of China, Sichuan Agricultural University, Chengdu, Sichuan 611130, China; Key Laboratory of Animal Disease-Resistant Nutrition of Sichuan Province, Sichuan Agricultural University, Chengdu, Sichuan 611130, China; School of Chemistry, Chemical Engineering and Biotechnology, Nanyang Technological University, Singapore 637371, Singapore; School of Chemistry, Chemical Engineering and Biotechnology, Nanyang Technological University, Singapore 637371, Singapore; College of Animal Science and Technology, Huazhong Agricultural University, Wuhan, Hubei 430070, China; Animal Nutrition Institute, Sichuan Agricultural University, Chengdu, Sichuan 611130, China; Key Laboratory for Animal Disease-Resistant Nutrition of the Ministry of Education of China, Sichuan Agricultural University, Chengdu, Sichuan 611130, China; Key Laboratory of Animal Disease-Resistant Nutrition of Sichuan Province, Sichuan Agricultural University, Chengdu, Sichuan 611130, China; Animal Nutrition Institute, Sichuan Agricultural University, Chengdu, Sichuan 611130, China; Key Laboratory for Animal Disease-Resistant Nutrition of the Ministry of Education of China, Sichuan Agricultural University, Chengdu, Sichuan 611130, China; Key Laboratory of Animal Disease-Resistant Nutrition of Sichuan Province, Sichuan Agricultural University, Chengdu, Sichuan 611130, China; Animal Nutrition Institute, Sichuan Agricultural University, Chengdu, Sichuan 611130, China; Key Laboratory for Animal Disease-Resistant Nutrition of the Ministry of Education of China, Sichuan Agricultural University, Chengdu, Sichuan 611130, China; Key Laboratory of Animal Disease-Resistant Nutrition of Sichuan Province, Sichuan Agricultural University, Chengdu, Sichuan 611130, China; Animal Nutrition Institute, Sichuan Agricultural University, Chengdu, Sichuan 611130, China; Key Laboratory for Animal Disease-Resistant Nutrition of the Ministry of Education of China, Sichuan Agricultural University, Chengdu, Sichuan 611130, China; Key Laboratory of Animal Disease-Resistant Nutrition of Sichuan Province, Sichuan Agricultural University, Chengdu, Sichuan 611130, China; Animal Nutrition Institute, Sichuan Agricultural University, Chengdu, Sichuan 611130, China; Key Laboratory for Animal Disease-Resistant Nutrition of the Ministry of Education of China, Sichuan Agricultural University, Chengdu, Sichuan 611130, China; Key Laboratory of Animal Disease-Resistant Nutrition of Sichuan Province, Sichuan Agricultural University, Chengdu, Sichuan 611130, China; Animal Nutrition Institute, Sichuan Agricultural University, Chengdu, Sichuan 611130, China; Key Laboratory for Animal Disease-Resistant Nutrition of the Ministry of Education of China, Sichuan Agricultural University, Chengdu, Sichuan 611130, China; Key Laboratory of Animal Disease-Resistant Nutrition of Sichuan Province, Sichuan Agricultural University, Chengdu, Sichuan 611130, China; State Key Laboratory of Food Science and Technology, Nanchang University, Nanchang, Jiangxi 330047, China; Animal Nutrition Institute, Sichuan Agricultural University, Chengdu, Sichuan 611130, China; Key Laboratory for Animal Disease-Resistant Nutrition of the Ministry of Education of China, Sichuan Agricultural University, Chengdu, Sichuan 611130, China; Key Laboratory of Animal Disease-Resistant Nutrition of Sichuan Province, Sichuan Agricultural University, Chengdu, Sichuan 611130, China; Animal Nutrition Institute, Sichuan Agricultural University, Chengdu, Sichuan 611130, China; Key Laboratory for Animal Disease-Resistant Nutrition of the Ministry of Education of China, Sichuan Agricultural University, Chengdu, Sichuan 611130, China; Key Laboratory of Animal Disease-Resistant Nutrition of Sichuan Province, Sichuan Agricultural University, Chengdu, Sichuan 611130, China; Animal Nutrition Institute, Sichuan Agricultural University, Chengdu, Sichuan 611130, China; Key Laboratory for Animal Disease-Resistant Nutrition of the Ministry of Education of China, Sichuan Agricultural University, Chengdu, Sichuan 611130, China; Key Laboratory of Animal Disease-Resistant Nutrition of Sichuan Province, Sichuan Agricultural University, Chengdu, Sichuan 611130, China

**Keywords:** time-restricted feeding, sexual dimorphism, estrogen receptor α, liver metabolism

## Abstract

Time-restricted feeding (TRF) is a dietary strategy used to prevent and treat obesity in both sexes. However, TRF affects liver metabolism differently in males and females, and the mechanisms behind these differences remain unclear. Our study reveals that during TRF, female livers are more likely to break down amino acids (AAs) to synthesize fats, while male livers significantly reduce fatty acid synthesis. The changes in the liver’s AA metabolic profile after gonadectomy suggest that estrogen signaling is crucial for regulating AA metabolism in females during TRF. Additionally, we demonstrate that hepatic estrogen receptor α (ERα)-mediated AA metabolism contributes to the sex-specific effects of TRF on liver metabolism. These findings offer new insights into the molecular mechanisms of TRF and its potential clinical application for treating fatty liver and other metabolic disorders. They also emphasize the need to consider sex differences when developing nutritional and pharmacological treatments for metabolic diseases in females.

## Introduction

The epidemic of overweight and obesity significantly increases the risk of developing type 2 diabetes, cardiovascular disease, nonalcoholic fatty liver disease (NAFLD), and even cancers, making it one of today’s most visible public health problems (https://www.who.int/health-topics/obesity). These conditions show notable gender disparities [1, 2]. For example, fertile women generally have a lower risk of developing type 2 diabetes and NAFLD compared to men and postmenopausal women [1, 2]. Achieving a negative energy balance through lifestyle modifications is a common strategy for managing obesity [3]. However, weight loss outcomes from lifestyle interventions display sex differences [4]. Men tend to lose more weight than women with non-pharmacological lifestyle interventions, including low-carbohydrate diets [5], low-protein diets [6], and exercise interventions [7].

Time-restricted feeding (TRF) is a dietary approach that includes various fasting protocols characterized by a daily fasting window [8, 9]. TRF has been reported to reduce weight gain and insulin resistance, and improve many of the metabolic abnormalities associated with obesity [10–13]. It is also important to note that, much like TRF, intermittent fasting has also been observed to have potentially negative effects on atherosclerosis and offspring metabolism [14, 15]. However, research on TRF has primarily focused on males, which significantly limits our understanding of the sex-specific mechanisms involved in TRF responses. TRF affects liver metabolism, including but not limited to bile acids, carbohydrates, lipids, and immunity, and it helps limit the onset and progression of NAFLD [16–19]. The liver, as a major metabolic organ, is influenced by gender [20–23]. In fertile females, hepatic fatty acid (FA) uptake, very low-density lipoprotein (LDL)-triglyceride (TG) formation and output, and FA oxidation occur at higher rates compared to males [24, 25]. However, further research is needed to confirm whether TRF’s beneficial effects on liver metabolism differ by sex. Liver estrogen receptor α (ERα), encoded by *Esr1*, shows gender differences in expression levels, being higher in females. The cycling activity of hepatic ERα provides females with essential metabolic flexibility [26, 27]. During short-term fasting, female livers tend to break down amino acids (AAs) to synthesize fat, which maintains the energy required for reproduction. This process is mainly mediated by liver ERα [28]. The transcriptional activity of hepatic ERα is closely linked to nutritional status [29, 30], but it is unclear whether the sexually dimorphic liver metabolism observed during TRF is mediated by liver ERα. Therefore, we hypothesize that gender differences in liver metabolism during TRF exist and hepatic ERα signaling may play a role.

Developing pharmacological approaches to address the obesity and diabetes epidemic requires a thorough understanding of the underlying mechanisms. Progress has been slow, partly due to the biological differences between males and females in these complex processes. In this study, we investigated whether liver ERα moderates the sexually dimorphic response to TRF through an unbiased transcriptomic analysis in both sexes. Our findings demonstrate that estrogen signaling mediates AA metabolism and contributes to the sex-dependent effects of TRF on liver metabolism via hepatic ERα. These results provide a solid foundation for ongoing translational research on TRF as a potential anti-diabetes treatment for both sexes.

## Results

### Estrogen loss mediates the metabolic benefits of TRF in the liver on a high-fat and high-sucrose (HFHS) diet

**Figure 1:**
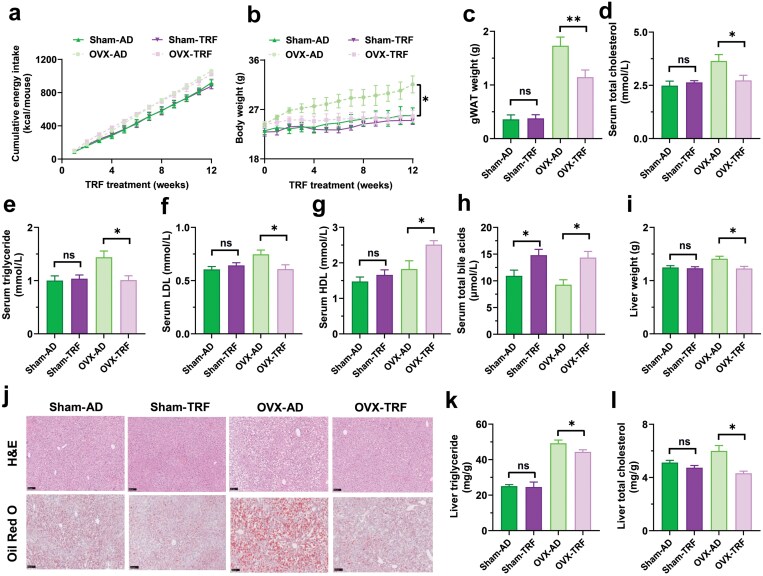
Estrogen loss mediates the metabolic benefits of TRF in the livers of female Sham mice and OVX mice fed on an HFHS diet. (a) Cumulative energy intake of mice per week. (b) Weekly body weight. (c) The gWAT weight. (d−h) Levels of serum TC (d), TG (e), LDL (f), HDL (g), and TBA (h). (i) Liver weight. (j) H&E and Oil Red O staining of the liver in mice. (k and l) Liver TG (k) and TC levels (l). Female Sham mice and OVX mice were fed *ad libitum* or with time-restricted access to food for 12 weeks. Data are presented as mean ± SEM. *n* = 8−10 per group. ^*^P < 0.05; ^**^P < 0.01.

To assess how estrogen deficiency affects the benefits of TRF, we compared the effects of TRF between sham-operated (Sham) mice and mice after gonadectomy. We divided female mice into four groups: Sham-AD, Sham-TRF, ovariectomy (OVX)-AD, and OVX-TRF, and subjected them to an HFHS diet for 12 weeks. Consistent with previous studies [31, 32], OVX increased cumulative energy intake, body weight, gondal white adipose tissue (gWAT) weight, serum total cholesterol (TC), TGs, and serum LDL compared to Sham mice (Fig. 1a–f). In contrast, TRF did not significantly alter cumulative energy intake, body weight, gWAT weight, serum TC, serum TG, serum LDL, or serum high-density lipoprotein (HDL) levels in Sham mice compared to *ad libitum* feeding (Fig. 1a–g). However, in OVX mice, TRF led to reductions in body weight, gWAT weight, serum TC, serum TG, and serum LDL levels, while increasing serum HDL levels (Fig. 1b–g). Serum total bile acids (TBAs) were increased in TRF mice, compared with AD mice, regardless of sham-operated or gonadectomy (Fig. 1h). Given that a Western diet tends to promote NAFLD, we also examined hepatic fat accumulation. Analysis of liver weight and histological staining with hematoxylin and eosin (H&E) and Oil Red O revealed that TRF reduced hepatic fat accumulation in OVX mice but not in Sham mice (Fig. 1i and j). This reduction was further supported by lower liver TC and TG levels (Fig. 1k and l). These findings underscore the significant role of systemic estrogen deficiency in mediating the metabolic benefits of TRF.

### Liver transcriptomic and metabolic profiling reveals an estrogen-biased response to TRF

**Figure 2:**
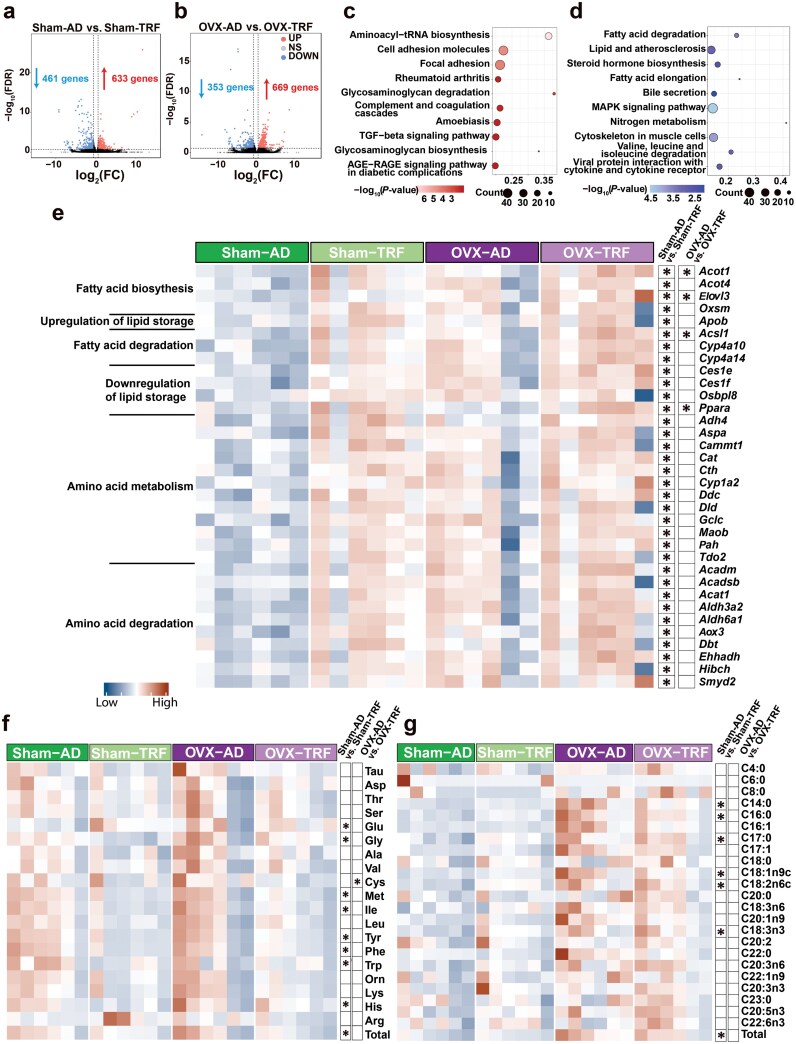
Liver transcriptomic and metabolic profiling reveals an estrogen-biased response to TRF. (a and b) Number of DEGs and volcano plot of DEGs in Sham mice (a) and OVX mice (b) based on mouse liver transcriptome sequencing during TRF. The DEGs were screened using the Deseq2 method, |Fold change (FC)| > 1.5, and false discovery rate (FDR) < 0.2. (c and d) KEGG analysis of DEGs using the Metascape online platform. Processes related to AA metabolism are highlighted in red (c), while those related to lipid metabolism are highlighted in blue (d). (e) Heatmaps of DEGs related to the upregulation of lipids, downregulation of lipids, AA metabolism, and AA degradation. (f) Liver AA content and total AA levels. (g) Liver FA content and total FA levels. Data are presented as mean ± SEM. *n* = 6 per group. ^*^P < 0.05.

To clarify the role of estrogen signaling in liver metabolism during TRF, we performed an unbiased transcriptomic analysis between AD and TRF mice in both Sham mice and mice after gonadectomy. This analysis showed that TRF upregulated 633 genes and downregulated 461 genes in Sham mice, while in OVX mice, 669 genes were upregulated and 353 genes were downregulated, compared with AD mice (Fig. 2a and b). Kyoto Encyclopedia of Genes and Genomes (KEGG) pathway analysis revealed that these differentially expressed genes (DEGs) were enriched in pathways related to AA metabolism (red) and fat metabolism (blue) (Fig. 2c and d). We focused on genes associated with AA and FA metabolism. In Sham mice, TRF generally increased the expression of genes involved in FA synthesis. In contrast, TRF led to a reduced number of upregulated genes associated with FA degradation in OVX mice (Fig. 2e). For AA metabolism, TRF specifically promoted the upregulation of several genes involved in AA metabolism and degradation in Sham mice, while OVX significantly diminished this TRF-induced upregulation (Fig. 2e).

Metabolic profiling further revealed that TRF decreased the levels of total AAs, including glutamic acid (Glu), glycine (Gly), methionine (Met), isoleucine (Ile), tyrosine (Tyr), phenylalanine (Phe), tryptophan (Trp), and histidine (His), in the livers of Sham mice, compared with Sham-AD mice. OVX effectively counteracted the effects of TRF on these AAs (Fig. 2f). Additionally, TRF increased the levels of FAs, including C14:0, C16:0, C17:0, C18:1n9c, C18:2n6c, and C18:3n3, in the livers of Sham mice, compared with Sham-AD mice (Fig. 2g). However, in OVX mice, the impact of TRF on these FAs was reduced (Fig. 2g). These results suggest that, in response to TRF, unlike OVX mice, females synthesized lipids, possibly by depleting the AA present in the liver.

### Hepatic ESR1 mediates sex differences in body weight change during TRF

**Figure 3:**
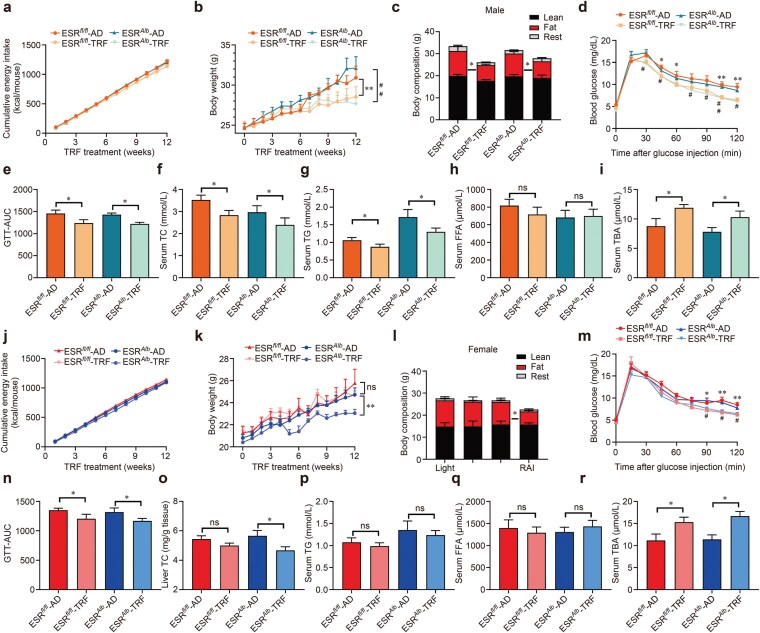
Hepatic ESR1 mediates sex differences in body weight changes during TRF. (a−c) Cumulative energy intake per week (a), weekly body weight (b), and body composition in male mice (c). (d and e) Changes of blood glucose levels (d) and the area under the curve (AUC) (e) in the GTT in male mice. (f−i) Serum levels of TC (f), TG (g), FFA (h), and TBA (i) in male mice. (i−l) Cumulative energy intake per week (j), weekly body weight (k), and body composition (l) in female mice. (m and n) Changes of blood glucose levels (m) and the AUC (n) in female mice. (o−r) Serum levels of TC (o), TG (p), FFA (q), and TBAs (r) in female mice. Data are presented as mean ± SEM. *n* = 8−10 per group. For ESR^fl/fl^-AD versus ESR^fl/fl^-TRF, ^*^P < 0.05; ^**^P < 0.01. For ESR^Alb^-AD versus ESR^Alb^-TRF, ^#^*P* < 0.05; ^##^*P* < 0.01.

Liver ERα is crucial for mediating sex-specific metabolic responses [28, 33]. To determine whether liver ERα affects sexually dimorphic responses to TRF, we analyzed the metabolic phenotypes of male and female mice lacking hepatic ERα (liver-specific *Esr1* knockout, ESRLKO). We first assessed the impact of TRF and hepatic *Esr1* knockout on metabolism in both sexes. Neither TRF nor hepatic *Esr1* knockout influenced food intake in male or female mice (Fig. 3a and j). In male mice, TRF positively affected metabolic outcomes, regardless of hepatic *Esr1* status. It reduced weight gain, especially in fat mass, induced by an HFHS diet (Fig. 3b and c), improved glucose tolerance (Fig. 3d and e), and lowered serum TC and TG levels (Fig. 3f and g), compared with AD mice. TRF did not alter serum free fatty acid (FFA) levels (Fig. 3h), however, TRF increased serum TBAs, compared with AD mice, regardless of hepatic *Esr1* knockout status (Fig. 3i).

In contrast, TRF did not affect HFHS-induced obesity in wild-type (WT) female mice (Fig. 3k). However, in the absence of hepatic ESR1, TRF mitigated obesity in these mice, especially in fat mass (Fig. 3k and l). TRF improved glucose tolerance in both WT and ESRLKO female mice (Fig. 3m and n). Consistent with the finding that TRF has no effects on serum TC and TG levels in fertile female mice (as shown in Fig. 1), TRF improved serum TC levels in ESRLKO female mice (Fig. 3o) but had no effect on serum TG levels (Fig. 3p). Nevertheless, serum FFA levels remained unchanged in both WT and ESRLKO female mice during TRF treatment (Fig. 3q). TRF increased serum TBAs compared with AD mice, regardless of hepatic *Esr1* knockout status (Fig. 3r).

**Figure 4:**
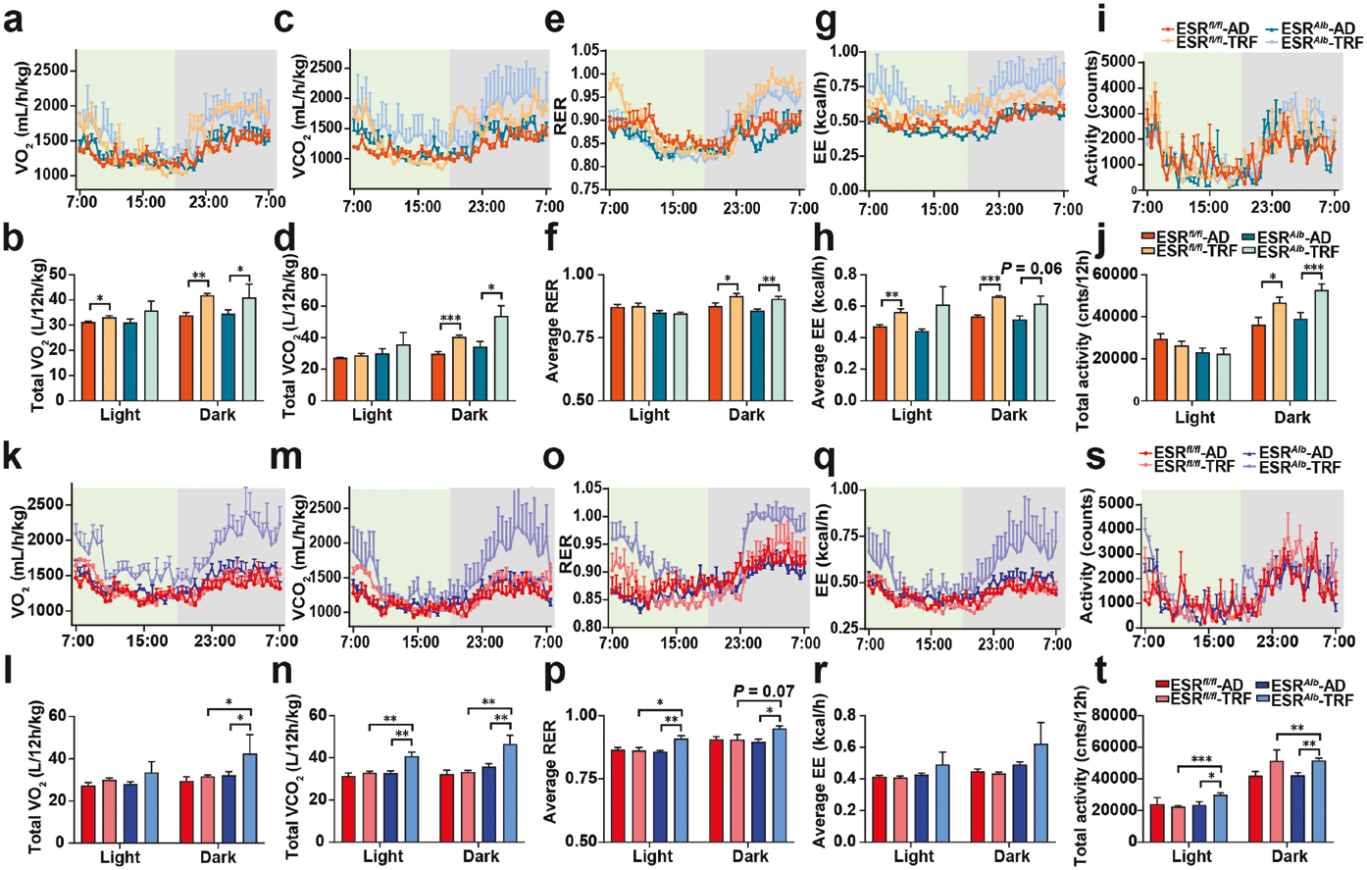
Hepatic ESR1 mediates sex differences in TRF-induced aerobic respiratory capacity improvement. (a−j) Oxygen consumption (a and b), carbon dioxide production (c and d), RER (e and f), EE (g and h), and locomotor activity (i and j) in male mice. (k−t) Oxygen consumption (k and l), carbon dioxide production (m and n), RER (o and p), EE (q and r), and locomotor activity (a and t) in female mice. Data are presented as mean ± SEM. *n* = 6 per group. ^*^P < 0.05; ^**^P < 0.01; ^***^P < 0.001.

We assessed the daily rhythm of substrate utilization using indirect calorimetry. In male mice, TRF increased oxygen consumption, carbon dioxide production, respiratory exchange ratio (RER), energy expenditure (EE), and locomotor activity during the dark phase, regardless of hepatic *Esr1* knockout status (Fig. 4a–j). TRF also elevated oxygen consumption and EE in WT male mice during the light phase (Fig. 4a and g). In female mice, increased oxygen consumption across the light/dark cycle occurred only after hepatic *Esr1* knockout (Fig. 4k and l). Additionally, TRF led to increased carbon dioxide production during the dark phase, elevated RER throughout the light/dark cycle, and higher locomotor activity during the entire light/dark cycle in female ESRLKO mice (Fig. 4m–t). However, TRF had no effect on EE in female mice (Fig. 4q and r). These findings highlight the crucial role of hepatic ESR1 in mediating the body weight and serum TC improvements induced by TRF in females.

### Hepatic ESR1 mediates sex differences in TRF’s protective effects against liver lipid accumulation

**Figure 5:**
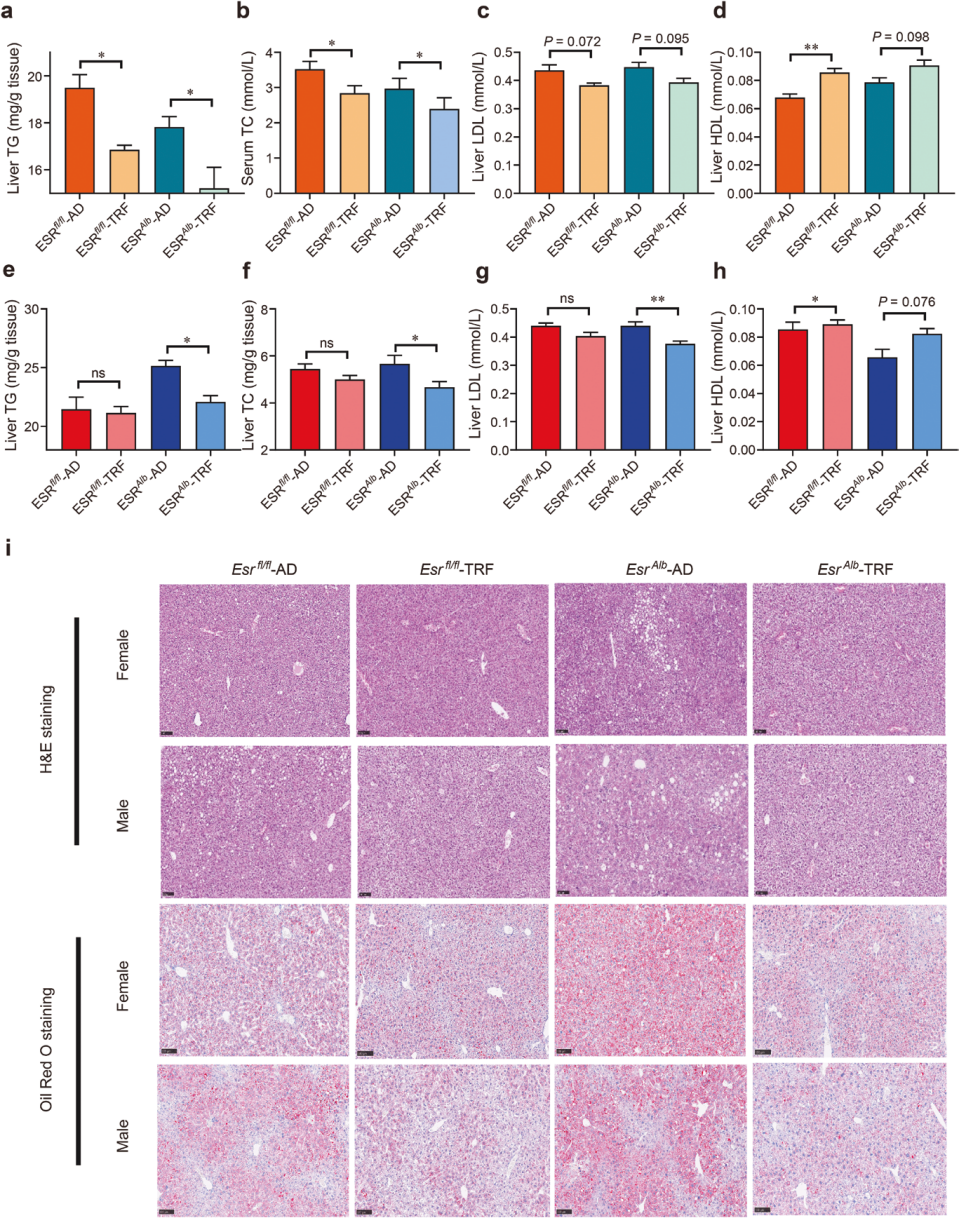
Hepatic ESR1 mediates sex differences in the protective effects of TRF against liver lipid accumulation. (a−d) Hepatic levels of TG (a), TC (b), LDL (c), and HDL (d) in male mice. (d−h) Liver levels of TG (e), TC (f), LDL (g), and HDL (h) in female mice. (i) H&E and Oil Red O staining of the livers in both male and female mice. Data are presented as mean ± SEM. *n* = 8−10 per group. ^*^P < 0.05; ^**^P < 0.01.

In male mice, TRF reduced hepatic levels of TG, TC, and LDL, while HDL levels increased, regardless of hepatic *Esr1* knockout status (Fig. 5a–d). In female mice, TRF did not affect TG, TC, LDL, or HDL levels in WT mice (Fig. 5e–h). However, hepatic *Esr1* knockout enhanced the beneficial effects of TRF on these lipid parameters (Fig. 5e–h), similar to the effects observed in male mice.

Histological analysis using H&E and Oil Red O staining of liver tissues revealed that TRF improved hepatic lipid accumulation in both WT and *Esr1*-knockout male mice (Fig. 5i). In female mice, TRF reduced hepatic lipid accumulation only when hepatic *Esr1* was absent (Fig. 5i). These findings indicate that hepatic ESR1 is crucial for mediating the protective effects of TRF against liver lipid accumulation, with notable sex differences in the presence or absence of this receptor.

### Hepatic transcriptome and metabolic profiling reveal sex differences in energy metabolism mediated by hepatic ESR1 in response to TRF

**Figure 6:**
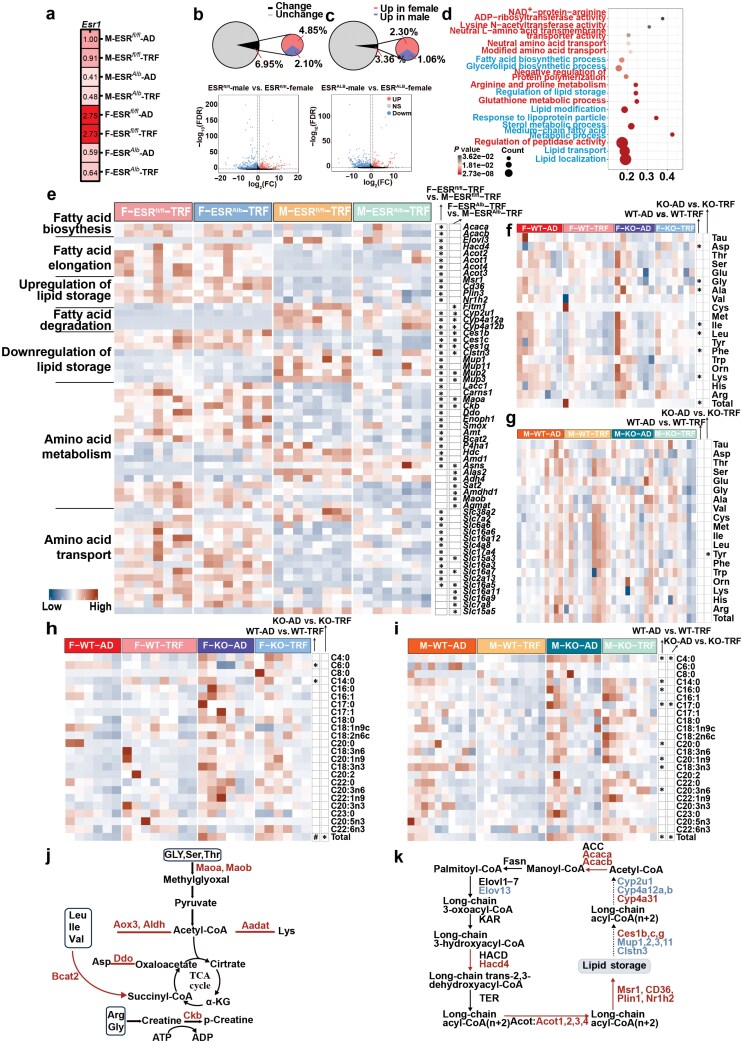
Hepatic transcriptome and metabolic profiling reveal sex differences in energy metabolism mediated by hepatic ESR1 in response to TRF. (a) Liver *Esr1* expression levels via transcriptomic analysis. (b and c) Number of DEGs and volcano plot of DEGs in male and female mice based on mouse liver transcriptome sequencing. The DEGs were screened using the Deseq2 method, |FC| > 1.5, and FDR < 0.01. (d) KEGG analysis of DEGs using the Metascape online platform. Processes related to AA metabolism are highlighted in red, while those related to lipid metabolism are highlighted in blue. (e) Heatmaps of DEGs related to the upregulation of lipids, downregulation of lipids, AA metabolism, and AA transport. *n* = 8 per group. (f and g) Liver AA content and total AA levels in female (f) and male mice (g). *n* = 7−9 per group. (h and i) Liver FA content and total FA levels in female (h) and male mice (i), *n* = 6−10 per group. (j and k) Comprehensive view of female liver metabolism in AA (j) and fat (k). Data are presented as mean ± SEM. ^*^P < 0.05; ^**^P < 0.01; ^#^0.05 < *P* < 0.1.

To investigate the mechanisms underlying liver metabolism patterns in response to TRF in both sexes, we performed a comprehensive transcriptomic analysis between female and male mice in both ESRLKO and WT mice. This analysis confirmed a reduction in *Esr1* gene expression in knockout mice (Fig. 6a). In WT mice, 6.95% of all liver-expressed genes showed sex differences, with 4.85% upregulated in females and 2.1% upregulated in males (Fig. 6b). Hepatic *Esr1* knockout reduced the proportion of sex-specific genes to 3.36% (2.30% upregulated in females and 1.06% in males), emphasizing the crucial role of hepatic ESR1 in modulating gender-specific gene expression (Fig. 6c).

KEGG pathway analysis revealed that the DEGs were primarily associated with AA and FA metabolism pathways (Fig. 6d). In TRF-treated WT mice, females exhibited upregulation of genes involved in FA biosynthesis and downregulation of those related to FA degradation compared to males (Fig. 6e). Hepatic *Esr1* knockout partially diminished these sex differences (Fig. 6e). Additionally, females showed upregulation of genes associated with AA metabolism and transport compared to males, with hepatic *Esr1* knockout similarly reducing these differences (Fig. 6e).

To further elucidate the metabolic reprogramming of the liver in response to TRF, we analyzed the profiles of AAs and FAs both in ESRLKO and WT mice. In female mice, TRF decreased the total AA content and levels of predominant AAs such as aspartic acid (Asp), Gly, alanine (Ala), Ile, leucine (Leu), Phe, and lysine (Lys). Hepatic *Esr1* knockout attenuated these effects (Fig. 6f). In contrast, TRF did not significantly affect AA levels in male mice (Fig. 6g). Regarding FAs, TRF increased the total FA content and levels of C6:0 and C14:0 in female mice, with hepatic *Esr1* knockout reversing these effects (Fig. 6h). In male mice, TRF reduced total FA levels and levels of several abundant FAs, regardless of hepatic *Esr1* status (Fig. 6i). Overall, these data suggest that TRF induces enhanced fat synthesis and AA degradation in the liver of female mice, with hepatic ESR1 playing a crucial role in modulating these sex-specific metabolic responses (Fig. 6j and k).

## Discussion

**Figure 7:**
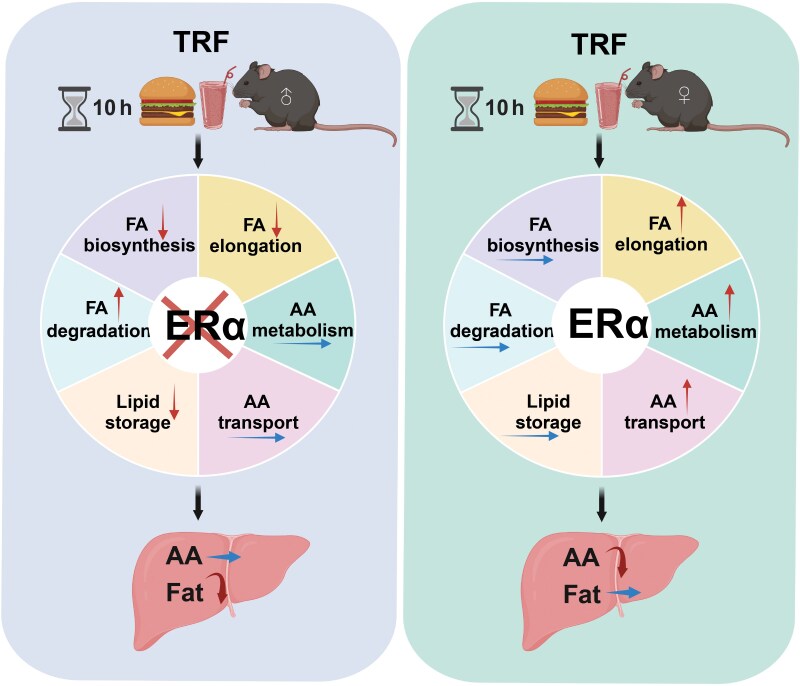
Working model of liver ERα mediating differences in metabolic pattern changes between sexes in response to TRF.

TRF is a nutritional intervention that benefits the treatment of obesity and metabolic syndrome in both sexes [10, 34, 35]. However, its effects on the liver differ between sexes [36, 37]. Our study shows that TRF causes the female liver to preferentially break down AAs for fat synthesis, while in males, TRF significantly reduces FA synthesis. We also discovered that hepatic ERα-mediated AA metabolism plays a role in the sex-dependent effects of TRF on liver metabolism (Fig. 7). These findings offer new insights into the molecular basis of the sexually dimorphic effects of TRF.

Natural selection has driven distinct metabolic adaptations to nutritional challenges in each sex, reflecting their different energy requirements for reproduction [20, 38, 39]. TRF is an effective intervention for preventing and treating diet-induced obesity and related metabolic diseases [8, 10, 12, 40, 41]. Although TRF improves insulin sensitivity, reduces inflammation, and extends lifespan in both sexes and across various ages [36, 42], it is also important to note that, much like TRF, intermittent fasting has also been observed to have potentially negative effects on atherosclerosis and offspring metabolism [14, 15].

TRF provides metabolic beneﬁts in a sex-dependent manner, including but not limited to body weight gain, adipose tissue inﬂammation, and liver metabolism [14, 18, 36]. Consistent with that, our study shows that TRF protects against HFHS diet-induced fatty liver in male mice, while this protective effect is less pronounced in females. Through hepatic transcriptome analysis, we provided a comprehensive overview of the liver’s response to TRF and highlighted sex-dependent differences in hepatic steatosis, which depend on the integrity of the female reproductive system. Females typically prioritize lipid synthesis over AA breakdown, preventing fat deposition in the liver, which partly explains their lower prevalence of fatty liver compared to males. After ovariectomy, we observed significant changes in liver AA and lipid metabolism, leading to fat accumulation in the liver. Notably, TRF induced a defeminization of AA metabolic profile in the liver after OVX, slowing down anabolic pathways and maintaining the AA pool. These observations emphasize the crucial role of estrogens in liver AA metabolism during TRF in females. Future studies should investigate whether androgen signaling in males significantly impacts the effects of TRF.

The liver regulates nutrient partitioning in a sex-dependent manner, with ERα playing a crucial “organizational” role in directing fuel selection and lipid handling [28, 29]. Our findings support this understanding, showing that hepatic ERα significantly influences the response to TRF by controlling liver AA metabolism. Under *ad libitum* conditions, male mice typically synthesize FAs and TGs, which they store in various organs, including the liver, muscle, and adipose tissues. During TRF, males suppress this synthesis pathway, whereas females continue to synthesize FAs through anabolic liver AAs. It is worth noting that we found abnormal body weight change in liver ERα knockout mice, despite not altering the overall trend in body weight changes. Consistent with previous studies [36], we found that TRF positively affected the body weight gain in male mice, but not in female mice. However, in the absence of hepatic ESR1 in female mice, TRF mitigated the body weight gain in these mice, suggesting that liver ESR1 mediates sex differences in TRF-induced body weight change. The phenotypes of glucose improvement and bile acid metabolism induced by TRF are not influenced by sex differences and not regulated by OVX or ERα deletion, suggesting that the effect of TRF outweighed the effect of sex and liver estrogen signaling on glucose intolerance and bile acid metabolism.

By using OVX and liver ERα knockout mice, we demonstrated that hepatic ERα-mediated AA metabolism contributes to the sex-dependent effects of TRF on liver metabolism. Our results suggest that circulating estrogens play a major role in maintaining hepatic sexual dimorphism [20, 28, 43, 44]. Notably, not all sex-dependent metabolic responses to TRF can be attributed to liver ERα. This observation may partly explain why females, both during and after the cessation of ovarian function, become more susceptible to hepatic and cardiovascular disorders.

In summary, our study reveals that the effects of TRF on liver metabolism are sexually dimorphic, with hepatic ERα-mediated AA metabolism playing a critical role in this process. These findings offer a novel molecular basis for the clinical application of TRF in treating fatty liver and other metabolic diseases. They may also have broad implications for the targeted use of nutritional and pharmacological interventions for metabolic disorders in both sexes.

## Limitations of the study

This study demonstrates that hepatic ERα mediates sex-specific metabolic adaptations to TRF. However, several limitations should be acknowledged. First, genes directly or indirectly regulated by hepatic ERα were not identified, leaving a gap in understanding the underlying molecular mechanisms. Future studies should employ unbiased metabolomics and lipidomics to comprehensively characterize metabolic and lipidomic shifts during TRF. Although body weight changes exhibited a consistent temporal pattern, the abnormal growth curves observed in Fig. 3 warrant further investigation. Similarly, while RER values displayed minor fluctuations, these variations remained within previously reported physiological ranges. Collectively, these limitations highlight the need for mechanistic studies to dissect ERα-driven pathways and validate the broader applicability of our findings.

## Materials and methods

### Animals

In this study, we conducted all animal procedures following the guidelines set by the Institutional Animal Care and Research Committee of Sichuan Agricultural University. We performed bilateral ovariectomies on 8-week-old mice. The mice were anesthetized with an intraperitoneal injection of pentobarbital sodium at a dose of 50 mg/kg body weight. We made longitudinal and bilateral abdominal incisions below the last lumbar vertebra. We carefully tied off and removed the ovary, oviduct, and the top of the fallopian tubes. After removing these tissues, we sutured the abdominal wall and skin to close the incisions. After a 2-week recovery period, the mice were used for experiments.

To generate ESRLKO mice, we mated ERα^loxp/loxp^ mice ob-tained from Jackson Laboratory with *Alb*-Cre mice to produce ESR^loxp+/−, Alb-Cre^ offspring. We then bred these mice with ESR^loxp/loxp^ mice to obtain ESR^loxp/loxp, Alb-Cre^ mice. We used the ESR^loxp/loxp^ littermates as controls in our experiments.

### Feeding schedule

During TRF treatment, the mice were fed with an HFHS diet, which consisted of 35% carbohydrate, 20% protein, and 45% fat (D12451), purchased from Research Diets (New Brunswick, NJ, USA). We randomly assigned all mice to either the *ad libitum* (AD) group or the TRF group, ensuring that each group started with equal weights. The TRF group followed a feeding schedule previously described [16]. Briefly, under TRF conditions, mice had unrestricted access to food for 10 h daily, from 10:00 p.m. to 8:00 a.m. on the second day. Every day, we transferred mice in the TRF group between cages with food and water and cages with only water. To control for handling, we also transferred mice in the AD group between feeding cages at the same time. We measured body weights and food intake weekly. The mice were maintained on a 12-h light/12-h dark cycle, with housing rooms kept at a temperature of 22−24°C.

### Metabolic profiling

We analyzed body composition using the EchoMRI-100 machine (Echo Medical Systems, Houston, TX, USA) to measure fat and lean mass in conscious mice. For metabolic analysis, we measured oxygen consumption, carbon dioxide production, RER, EE, and locomotor activity simultaneously in individually housed mice using a comprehensive laboratory animal monitoring system (Columbus Instruments). We acclimatized the mice to the monitoring system for 2 days, and then collected data over the subsequent 2−3 days. We maintained consistent light and feeding conditions in the monitoring environment, matching those in the regular housing cages, to ensure the reliability of the collected data.

### Glucose tolerance test (GTT)

After overnight fasting, we administered an intraperitoneal injection of d-glucose at a dose of 1 g/kg body weight to conduct a GTT. We measured blood glucose levels at several time points: 0, 15, 30, 45, 60, 75, 90, 105, 115, and 120 min after injection. We collected these measurements using tail vein blood and glucose test strips from Roche Diagnostics.

### Sample collection

After 12 weeks of treatment, we euthanized the mice using carbon dioxide. We collected heart blood, liver, and fat samples at 8:00 a.m. We centrifuged the blood samples for 15 min at 3000 *g* to obtain serum, which we stored at −20°C for future analysis. We fixed a portion of the left lobe of the liver and adipose tissue in 4% paraformaldehyde for histological examination. We froze the remaining tissues in liquid nitrogen and stored them at −80°C for future analyses.

### Serum biochemistry

We measured serum levels of TGs, FFA, TC, HDL, LDL, and TBA using an automatic biochemical analyzer (Model 3100, Hitachi, Tokyo, Japan).

### Histological staining

We preserved liver samples in a 4% paraformaldehyde solution and embedded them in both paraffin and optimal cutting temperature (OCT) embedding media. We sectioned paraffin-embedded liver samples at 3-μm thickness and stained them with H&E to evaluate general histology. Snap-frozen liver samples embedded in OCT were sectioned at 8 μm and stained with Oil Red O to assess lipid content. We similarly preserved subcutaneous adipose tissue and gWAT in a 4% paraformaldehyde solution and embedded them in paraffin. We sectioned these paraffin-embedded adipose samples at 3-μm thickness and stained them with H&E. We imaged the slides using a light microscope. We quantified adipocyte cell size (area in μm²) using Adiposoft ImageJ. We quantified three sections per mouse, with each section covering an approximate area of 2 mm^2^ and containing about 300 adipocytes, using samples from six mice per group.

### Liver AA analysis

To determine the AA composition and content of liver samples, we used a fully automated AA analyzer (Hitachi, LA8080, Tokyo, Japan). The procedure was as follows: we added 0.2 mol/L HCl to lyophilized liver samples and thoroughly homogenized the mixture. We then centrifuged the homogenized sample and aspirated the supernatant. We precipitated the protein using the sulfosalicylic acid assay, vortexed the mixture, and allowed it to stand for half an hour at 4°C. We centrifuged the mixture again and aspirated the supernatant. We filtered the supernatant through a micro-filter and transferred it to an onboard vial for analysis by the AA analyzer.

### Liver FA analysis

We determined the FA contents of various carbon chains in liver samples using a gas chromatograph (Shimadzu, 2010 Plus, Kyoto, Japan). We weighed and homogenized lyophilized liver tissue in a chloroform:methanol:water (8:4:3) mixture. We transferred the homogenate to a glass centrifuge tube, vortexed it, and then centrifuged it. We transferred the chloroform layer to a new glass centrifuge tube and evaporated it in a vacuum-drying oven, yielding a fatty acid glycerol ester mixture. We added KOH-CH_3_OH solution (0.5 mol/L) to this mixture, vortexed it, and placed it in a 50°C water bath until the oil droplets disappeared, completing the saponification process and obtaining an FFA mixture. We cooled the mixture in an ice-water bath, treated it with BF_3_-CH_3_OH solution (14%), vortexed it, and refluxed it at 80°C for methyl esterification. After cooling it to room temperature, we added *n*-hexane and saturated sodium chloride solution, vortexed the mixture, and centrifuged it. We then transferred the upper *n*-hexane layer to vials for analysis by the gas chromatograph.

### RNA sequencing

We sequenced RNA libraries using the Illumina NovaSeq™ 6000 platform (LC Biotechnology Co., Ltd., Hangzhou, China). We isolated and purified total RNA with TRIzol (Thermo Fisher, cat. 15596018) according to the manufacturer’s protocol. We assessed RNA quantity and purity using NanoDrop ND-1000 (NanoDrop, Wilmington, DE, USA) and evaluated RNA integrity with Bioanalyzer 2100 (Agilent, CA, USA). We deemed RNA samples suitable for further processing if they met the criteria of concentration > 50 ng/μL, RNA integrity number (RIN) value > 7.0, and total RNA amount > 1 μg. We selectively captured poly(A)-tailed mRNA through two rounds of purification using oligo(dT) magnetic beads (Dynabeads Oligo(dT), cat. 25-61005, Thermo Fisher, USA). We then fragmented the captured mRNA with the NEBNext^®^ Magnesium RNA Fragmentation Module (cat. E6150S, USA) at 94°C for 5−7 min. We reverse-transcribed this fragmented RNA to cDNA using SuperScript™ II Reverse Transcriptase (Invitrogen, cat. 1896649, CA, USA).

Next, we used *Escherichia coli* DNA Polymerase I (NEB, cat. M0209, USA) and RNase H (NEB, cat. M0297, USA) to convert the DNA-RNA hybrids into double-stranded DNA. We treated the double-stranded DNA with dUTP Solution (Thermo Fisher, cat. R0133, CA, USA) to modify the ends and facilitate the addition of an A base at each end, enabling ligation to T-tailed adapters. We screened and purified fragment sizes using magnetic beads. We digested the second strand of cDNA with UDG enzyme (NEB, cat. M0280, MA, USA) and prepared the library by PCR amplification: pre-denaturation at 95°C for 3 min, followed by 8 cycles of denaturation at 98°C for 15 s, annealing at 60°C for 15 s, and extension at 72°C for 30 s, with a final extension at 72°C for 5 min. We sequenced the resulting strand-specific library fragments, which were around 300 ± 50 bp in size, using the Illumina NovaSeq™ 6000 platform with paired-end 150 bp (PE150) reads.

### Transcriptomic data analysis

We analyzed transcriptomic data to identify differential gene expression using the OmicShare platform (http://www.omicshare.com/tools) in combination with DESeq2 software. For the liver *Esr1* knockout experiments, we defined genes as differentially expressed if they met the criteria of |Fold Change (FC)| > 1.5 and false discovery rate (FDR) < 0.01. In contrast, for the OVX experiments, we used criteria of |FC| > 1.5 and FDR < 0.2 to determine differential expression. We generated heat maps and performed principal component analysis using the OmicShare platform. Additionally, we conducted KEGG and Gene Ontology (GO) enrichment analyses using the Metascape online tool (https://metascape.org/gp/index.html).

### Statistical analysis

We analyzed the data using GraphPad Prism 8 software (GraphPad Software, La Jolla, CA, USA). We employed a two-way analysis of variance (ANOVA) test, followed by Tukey’s test for multiple comparisons among groups. Results are presented as mean values ± standard error of the mean (SEM). We determined statistical significance at a threshold of *P* < 0.05.

## Data Availability

All data necessary to support the conclusions of this study is included in the paper. The datasets generated and/or analyzed during the current study are available upon reasonable request from the corresponding author.
